# Hexagonal Rare Earth-Iron Mixed Oxides (REFeO_3_): Crystal Structure, Synthesis, and Catalytic Properties

**DOI:** 10.3389/fchem.2019.00008

**Published:** 2019-01-28

**Authors:** Saburo Hosokawa

**Affiliations:** ^1^Elements Strategy Initiative for Catalysts and Batteries, Kyoto University, Kyoto, Japan; ^2^Department of Molecular Engineering, Graduate School of Engineering, Kyoto University, Kyoto, Japan

**Keywords:** solvothermal method, coprecipitation method, catalyst, perovskite structure, hexagonal structure

## Abstract

The rare earth-iron mixed oxide (REFeO_3_) is an attractive material in fields such as electronic, magnetic, and catalytic research. Generally, orthorhombic REFeO_3_ (*o*-REFeO_3_) with a perovskite structure is better known than hexagonal REFeO_3_ (*h*-REFeO_3_), because *o-*REFeO_3_ is thermodynamically stable for all RE elements. However, *h*-REFeO_3_ has a very interesting crystal structure in which a RE and Fe layer are alternately stacked along the *c*-axis in the unit cell; nevertheless, synthesis of the *h*-REFeO_3_ belonging to metastable phase can be problematic. Fortunately, solution-based synthetic methods like solvothermal or coprecipitation synthesis have recently enabled the selective synthesis of *h*-REFeO_3_ and *o*-REFeO_3_ with comparative ease. Although the electronic and magnetic properties of *h*-REFeO_3_ have typically been evaluated, recent research has also revealed excellent catalytic properties that enable environmental cleanup reactions such as hydrocarbon or CO oxidation. This mini-review introduces a synthetic method for controlling the crystal structure between orthorhombic and hexagonal REFeO_3_ and the catalytic performance of *h*-REFeO_3_-based materials.

## Introduction

REFeO_3_ (RE: rare earth) with a RE/Fe ratio of 1 are largely relevant to the perovskite structure, a structure in which RE ions are basically replaced with 1/4 of cubic close-packed oxygen ions, and Fe ions occupy all octahedral gaps formed from the remaining oxygen ions. REFeO_3_ having a perovskite structure have been applied in various fields from electronic devices to catalysts. On the other hand, hexagonal REFeO_3_ is known to exist as a metastable phase, but the synthetic method is very complex. Interestingly, the crystal structure has a unique coordination state for Fe^3+^ ion and is different from that of the perovskite-type REFeO_3_; that is, Fe^3+^ ions in hexagonal structure have trigonal bipyramidal coordination, although Fe^3+^ ions in α-Fe_2_O_3_, Fe_3_O_4_, or perovskite-type REFeO_3_ generally prefer octahedral or tetrahedral coordination. Therefore, hexagonal structure has a potential which provokes an interesting property for the above applied fields. In particular, novel catalysts composed on abundant base metal as Fe ion have been recently desired to replace the use of precious metals, such as Pt or Pd; the catalyst design based on a unique crystal structure is considered to be necessary to dramatically improve the catalytic performance of Fe-based material.

**Figure 1:**
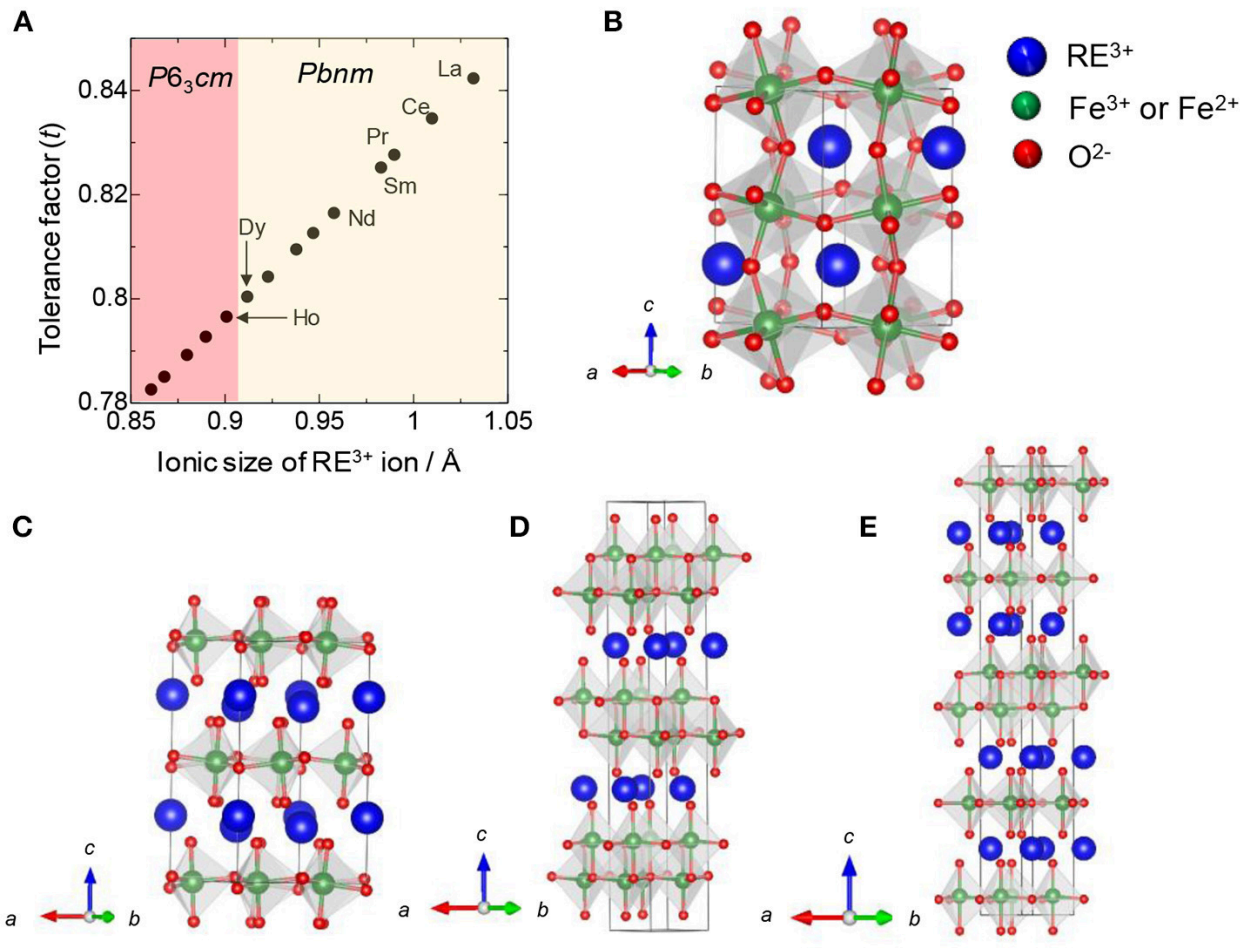
**(A)** Tolerance factor and stable crystal structure against ionic size of RE in REMnO_3_, **(B)** Orthorhombic REFeO_3_ (*o*-REFeO_3_) or *o*-REMnO_3_ with the space group of *Pbnm*, **(C)** Hexagonal REFeO_3_ (*h*-REFeO_3_) or *h*-REMnO_3_ with the space group of *P*6_3_*cm*, **(D)** REFe_2_O_4_, and **(E)** RE_2_Fe_3_O_7_.

First, the relationship between RE elements and hexagonal structure is introduced, based on a general perovskite structure. Considering that each ion is in contact within the perovskite structure, the following equation is true: (r_A_ + r_O_) = √2(r_B_ + r_O_), where r_A_, r_B_, and r_O_ represent the ionic radii of the A cation as RE ion, B cation as Fe ion, and oxygen ion, respectively. Therefore, the perovskite structure is generally considered to be a stable phase, when the tolerance factor, *t* = [(r_A_ + r_O_)]/[√2(r_B_ + r_O_)] is close to 1 (Schneider et al., 1961; Woodward, 1997; Li et al., 2004). Regarding the relationship between RE elements and tradition metal ions, the tolerance factor decreases with decreasing ionic radius of the RE element (Kumar et al., 2011); that is, the perovskite structure becomes less stable. However, for REFeO_3_, the orthorhombic phase is thermodynamically stable for all RE elements, and the trend is different from a general trend observed in REMnO_3_ (Figure 1A); orthorhombic REMnO_3_ (*o*-REMnO_3_) with a perovskite structure and the space group of *Pbnm* is preferentially formed for RE elements with large ionic sizes (La–Dy), while hexagonal REMnO_3_ (*h*-REMnO_3_) with *P*6_3_*cm* is obtained for RE elements with small ionic sizes (Ho–Lu and Y) (Figures 1B,C). As the ionic size of an Fe^3+^ ion is almost the same as that of an Mn^3+^ ion, the difference in the electronic configuration of d orbitals between Fe^3+^ and Mn^3+^ ions may contribute to the stability of the hexagonal phase. In other words, because *h*-REFeO_3_ is a thermodynamically unstable phase, it is relatively poorly studied compared with *o*-REFeO_3_.

By controlling the synthetic route, *h*-REFeO_3_ composed from RE ions with a smaller ionic radius than Er or Y have been obtained. The space group of *h*-REFeO_3_ has been reported as *P*6_3_*cm* with a YMnO_3_ structure type or *P*6_3_/*mmc* with a YAlO_3_ structure type (Li et al., 2008; Kumar et al., 2009; Magome et al., 2010). The crystal structure belonging to *P*6_3_*cm* is very similar to that to *P*6_3_/*mmc*, except for a slightly more distorted crystal structure in the former than in the latter. Actually, the oxygen layer has a zigzag structure in the *P*6_3_*cm* structure. In either space group, *h*-REFeO_3_ has a unique crystal structure other than the coordination state of Fe^3+^ ion; RE and Fe layers are alternately stacked along the *c*-axis. REFe_2_O_4_ or RE_2_Fe_3_O_7_, represented by the chemical formula RE_n_Fe_n+1_O_(3n+1)_, are known as hexagonal phase-related materials (Malaman et al., 1976; Qin et al., 2009; Bourgeois et al., 2012; Lafuerza et al., 2014), and the coordination environment of Fe ions is a trigonal bipyramidal structure similar to the structure of *h*-REFeO_3_. REFe_2_O_4_ has a hexagonal layer structure with the space group of *R*$\bar{{}^{\text{3}}}$*m*, which is formed by the alternate stacking of two layers with compositions of REO_3/2_ and Fe^2+^Fe^3+^O_5/2_ (Figure 1D). The Fe layer consists of two triangular sheets of corner-sharing FeO_5_ trigonal bipyramids. RE_2_Fe_3_O_7_ can be transcribed to REFe_2_O_4_•(REFeO_3_), and the structures of the REFe_2_O_4_•(REFeO_3_)_n_ phases are described by alternately stacking REFe_2_O_4_ and hexagonal REFeO_3_ blocks along the *c*-axis direction (Figure 1E).

When *h*-REFeO_3_ is viewed as an applied material, its layered structure composed of RE and Fe layers along the *c*-axis is very attractive for multiple purposes, from electric to catalytic applications. In fact, LuFe_2_O_4+x_ has shown excellent oxygen storage properties because its hexagonal-related structure has unique structural flexibility accompanied by “topotactic transition” during the oxygen intercalation/de-intercalation process (Hervieu et al., 2014). In this transformation, the original cation ordering in the hexagonal structure is maintained without any intermixing between RE and Fe ions. This mini-review therefore presents the method for controlling the crystal structure of *h*-REFeO_3_ and *o*-REFeO_3_ and discusses the catalytic properties of *h*-REFeO_3_.

## Solvothermal Method

Because *h*-REFeO_3_ is a metastable phase, a unique synthesis method is typically required. Among existing methods, a solvothermal method is effective for controlling the hexagonal and orthorhombic phases. In this method, synthesis of inorganic material is achieved by reaction in a liquid medium at high temperatures in a closed vessel (autoclave) (Demazeau, 1999; Cushing et al., 2004). Therefore, the traditional solvothermal method is a hydrothermal method in which water is used as the solvent. Since the last three decades, alcohol or glycol has been used as a solvent in the solvothermal method (Fanelli and Burlew, 1986; Das et al., 2008), with which various metal oxides or mixed oxides with unique morphology or ultrafine nanoparticles have been reported.

**Figure 2:**
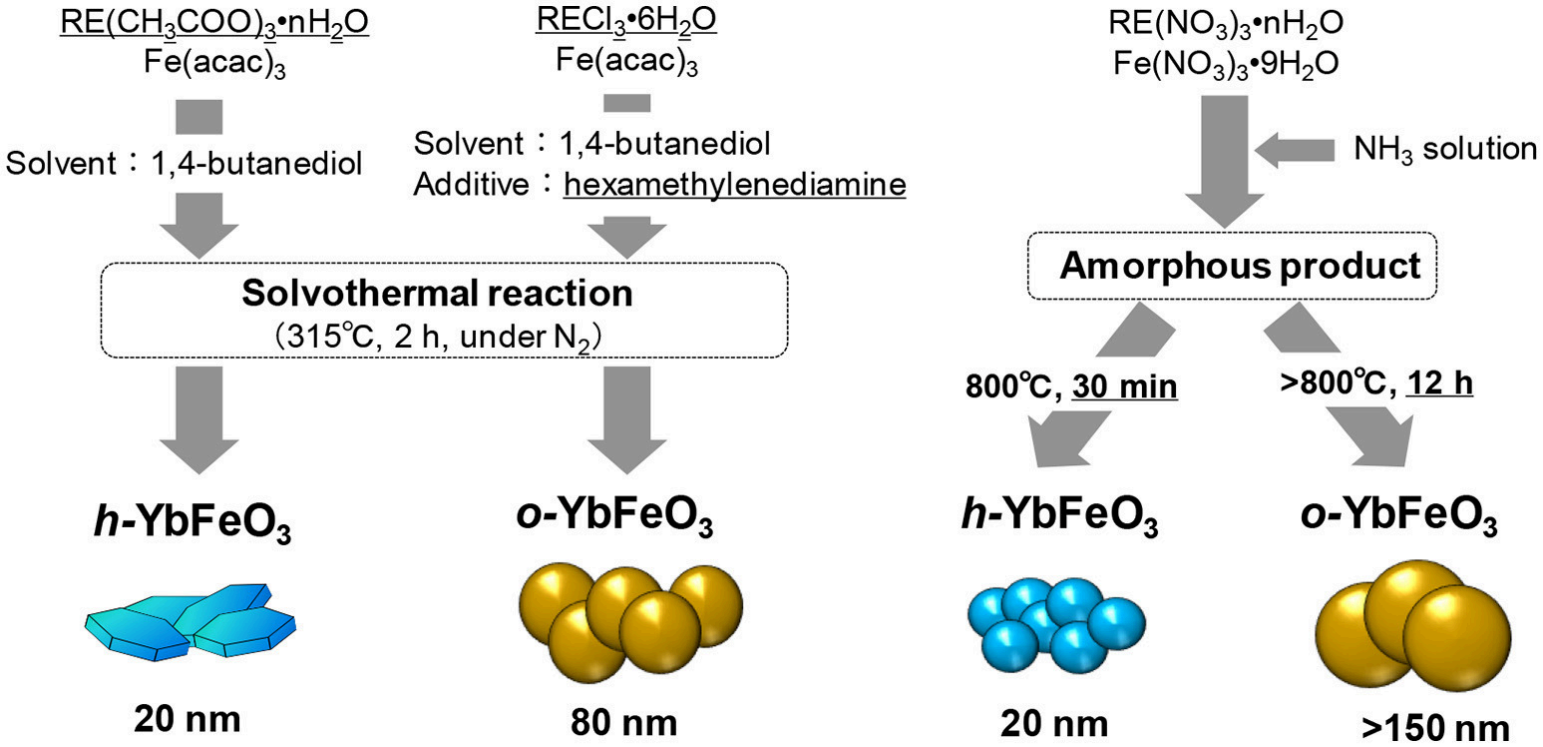
Flow chart showing crystal structure control utilizing the solvothermal method **(Left)** and coprecipitation method **(Right)**.

Inoue et al. found that *h*-REFeO_3_ nanoparticles composed of RE with a smaller ionic radius than Er can be obtained through a non-aqueous solvothermal reaction of RE acetate with Fe acetylacetonate in 1,4-butanediol at 300°C (Figure 2; Inoue et al., 1997). It should be noted that crystal growth in the *c*-axis direction in *h*-REFeO_3_ is drastically suppressed during solvothermal synthesis, resulting in the formation of nanoparticles with a particle size of approximately 20 nm and a well-formed hexagonal plate-like morphology. It was found that *h*-YbFeO_3_ and *o*-YbFeO_3_ can be controlled by the choice of reaction conditions and the starting material for the solvothermal reaction (Hosokawa et al., 2009). Interestingly, when RE chloride (RE = Sm-Yb) and Fe acetylacetonate are used for the solvothermal reaction in 1,4-butanediol in the presence of hexamethylenediamine, monodisperse particles of *o*-REFeO_3_ (crystallite size: about 80 nm) are formed. It is suggested that *o*-YbFeO_3_ has a larger crystallite size than solvothermally-synthesized *h*-YbFeO_3_, which indicates that crystal nucleation is more difficult for *o*-YbFeO_3_ than *h*-YbFeO_3_.

High-resolution transmission electron microscopy (TEM) of Yb_3_Fe_4_O_10_ revealed that three or more YbFeO_3_ layers are inserted between the YbFe_2_O_4_ layers (Matsui et al., 1979), suggesting that the REFe_2_O_4_ phase with Fe^3+^ and Fe^2+^ species may act as a nucleus for *h*-REFeO_3_. The result indicates that the existence of Fe^2+^ ions plays an important role in the formation of *h*-REFeO_3_. The solvothermal reaction in 1,4-butanediol causes reductive conditions because of the reducing ability of glycol; that is, Fe^2+^ species formed in the reaction system seem to contribute to the formation of *h*-REFeO_3_. On the other hand, because the incorporation of amine into the solvothermal reaction suppresses the reduction from Fe^3+^ to Fe^2+^ ions owing to the chelating ability of hexamethylenediamine, *o*-YbFeO_3_ must be obtained. In addition, the reducibility of Fe^3+^ species is unsurprisingly also considered to depend on whether the ligand of the starting material is acetate or chloride.

This non-aqueous solvothermal method enables the crystallization of REFeO_3_ nanoparticles below 100 nm without calcination. Therefore, it is promising to control the crystal structure, shape, and particle size by adjusting the additives and solvent in the solvothermal system.

## Coprecipitation and Sol-gel Method

General synthetic routes such as the coprecipitation method inevitably involve a calcination process for the synthesis of metal oxide materials, and it is difficult to synthesize the metastable phase. Therefore, *h*-REFeO_3_ is often synthesized by optimizing the crystallization process. For example, *h*-REFeO_3_ is synthesized by introducing solutions containing metal salts into a high-temperature inductively coupled plasma (ICP) (Mizoguchi et al., 1996). An instant crystallization under the ICP atmosphere yields *h*-REFeO_3_ nanoparticles sized 20–50 nm. Kumar et al. reported that the hexagonal phase is synthesized by rapidly cooling a melt of the composition REFeO_3_ under controlled oxygen pressure (Vijaya Kumar et al., 2008). This method requires special equipment in which the precursor is suspended on a nozzle fitted to an oxygen gas jet, thereby preventing heterogeneous nucleation that occurs on the walls of the container. As a result, *h*-LuFeO_3_ with high crystallinity is obtained under an oxygen partial pressure of 1 × 10^5^ Pa.

On the other hand, in the case of *h*-REFeO_3_ synthesis from an amorphous precursor obtained by coprecipitation method in which ammonia water is added immediately to an aqueous solution containing RE and Fe ions (Nishimura et al., 2013), *h*-REFeO_3_ for RE ions with a smaller ionic radius than Er can be easily obtained under precisely controlled calcination conditions. For an amorphous precursor with the composition of YbFeO_3_, an exothermic peak without weight change is observed in the temperature range slightly below 800°C, by thermal gravimetric-differential thermal analysis. Therefore, the sample calcined in air at 700°C for 30 min is amorphous, and the formation of pure-phase *h*-YbFeO_3_ is confirmed by calcining the amorphous product at 800°C for 30 min (Figure 2). Note that, when the retention time at 800°C is extended to 12 h, *o*-YbFeO_3_ is obtained as the main product. These results strongly suggest that the exothermic peak is attributed not only to crystallization from an amorphous to hexagonal structure but also a phase transition from a hexagonal structure to orthorhombic structure. In other words, the hexagonal and orthorhombic phase can be kinetically classified by adjusting the heat retention time in the calcination process. In this manner, because a subtle change in calcination conditions leads to the formation of *h*-REFeO_3_ from an amorphous product, the synthesis of *h*-REFeO_3_ is much more difficult than that of *o*-REFeO_3_.

Similar crystallization behavior is also observed for a precursor containing organic species synthesized by a citric acid-assisted sol-gel process (Zhang et al., 2012). For an amorphous precursor with the composition of YFeO_3_, the crystallization temperature of *h*-YFeO_3_ is 700°C. This is lower than that of *h*-YbFeO_3_ (800°C); therefore, the crystallization temperature depends on the ionic radius of RE ions. Interestingly, doping Pd on *h*-YFeO_3_ via the sol-gel method stabilizes the hexagonal phase, and the phase transformation to the orthorhombic phase hardly proceeds even by calcination at 1,000°C (Li et al., 2008).

*h*-REFeO_3_, which is obtained by the calcination of a precursor synthesized by the solution method, often comprises fine particles with a crystallite diameter below 50 nm. On the other hand, *o*-REFeO_3_, which is obtained by phase transition from *h*-REFeO_3_, has a much larger particle size than *h*-REFeO_3_. For example, the particle size of *h*-YbFeO_3_ synthesized by the coprecipitation method is approximately 20 nm, whereas that of *o*-YbFeO_3_ is above 150 nm. A nucleation and growth mechanisms has been reported to proceed in a phase transition from γ-Al_2_O_3_ or θ-Al_2_O_3_ to α-Al_2_O_3_ (Dynys and Halloran, 1982; Bagwell et al., 2001): that is, rapid crystal growth of α-Al_2_O_3_ simultaneously progresses with the formation of crystal nuclei of α-Al_2_O_3_, resulting in the formation of α-Al_2_O_3_ with a very large particle size. Similar to the phenomena for α-Al_2_O_3_, crystal nucleation and rapid crystal growth of *o*-REFeO_3_ must proceed during the phase transition from *h*-REFeO_3_ to *o*-REFeO_3_. These results imply that *h*-REFeO_3_ nanoparticles can be synthesized by the coprecipitation or sol-gel method, but *o*-REFeO_3_ with a particle size below 100 nm is difficult to synthesize for RE ions with a smaller ionic radius than Er.

## Catalytic Performance of *h*-REFeO_3_

*o*-REFeO_3_ has been applied as a catalyst material in various fields including the purification of volatile organic compound etc. (Ciambelli et al., 2001; Barbero et al., 2006). Applications of *h*-REFeO_3_ have been rare, except for in magnetic or electronic fields, until about 15 years ago. However, recent applications have emerged in the field of catalysts (Kurzman et al., 2011; Ismael et al., 2017). For example, *h*-YFeO_3_ has been reported to show a higher catalytic activity than *o*-YFeO_3_ for the photodecomposition of methyl orange under visible light irradiation (> 420 nm) (Zhang et al., 2012). As *h*-YFeO_3_ (1.94 eV) has a narrower band gap than *o*-YFeO_3_ (2.43 eV), the band structure of *h*-YFeO_3_ may be contributed to the high catalytic activity. *h*-YFeO_3_ with the unique band structure is also demonstrated to be suitable as a photoanode for solar water splitting (Guo et al., 2017).

The catalytic performance of *h*-REFeO_3_ as an oxidation catalyst has actively been investigated. When CH_4_ oxidations over *h*-YbFeO_3_ and *o*-YbFeO_3_ obtained by the solvothermal reaction are evaluated (Hosokawa et al., 2011), the *h*-YbFeO_3_ catalysts shows extremely high combustion activity compared with *o*-YbFeO_3_. The *T*_50_ value, at which the catalyst exhibits 50% CH_4_ conversion, is 475°C for *h*-YbFeO_3_, while that for *o*-YbFeO_3_ 550°C. The surface area of the *o*-YbFeO_3_ catalyst significantly decreases during calcination at 800°C in catalyst preparation; that is, the surface area of *o*-YbFeO_3_ synthesized by the solvothermal method is 26 m^2^g^−1^ and that of *o*-YbFeO_3_ after calcination is 6 m^2^g^−1^. Interestingly, the surface area of the *h*-YbFeO_3_ catalyst (approximately 30 m^2^g^−1^) does not change significantly during the calcination, and the well-formed hexagonal plate shape is maintained. This result suggests that the surface energy of *o*-YbFeO_3_ is higher than that of *h*-YbFeO_3_. In other words, because *h*-YbFeO_3_ synthesized by the solvothermal method has high thermal stability, high catalytic activity must be achieved.

The *h*-YFe_1−x_Pd_x_O_3−δ_ catalyst synthesized by the sol-gel method has been reported to show high catalytic activity (*T*_50_ = 100°C) for CO oxidation under excess O_2_ concentration (1,000 ppm CO and 10% O_2_), comparable to that of a Pd/Al_2_O_3_ catalyst with high surface area (Li et al., 2008). The ionic Pd species in the hexagonal lattice contributes to the catalytic activity. Lu et al. also reported that a Pd catalyst supported on *h*-YFeO_3_ is more effective for CH_4_ oxidation than Pd/*h*-YMnO_3_ or Pd/*o*-LaFeO_3_, and that the Pd/YFeO_3_ catalyst maintains a high catalytic activity for CH_4_ oxidation even after aging treatment at 900°C despite the phase transformation to *o*-YFeO_3_ (Lu et al., 2014a,b, 2015). In these cases, as the Pd species is adequately anchored with the *h*-YFeO_3_ support, the catalyst seems to have high catalytic activity.

Furthermore, to develop a noble-metal free *h*-YbFeO_3_-based catalyst with high catalytic activity, transition metal-modified *h*-YbFeO_3_ catalysts are synthesized by solvothermal methods (Hosokawa et al., 2016). Mn modification by the solvothermal method dramatically improves the catalytic activity of *h*-YbFeO_3_ itself for CO oxidation in which a reaction gas composed of 5,000 ppm CO and 5,000 ppm O_2_ is introduced on catalyst bed. The catalytic activity (*T*_50_ = 120°C) of Mn-modified *h*-YbFeO_3_ (Mn-*h*-YbFeO_3_) with the composition of YbFe_0.6_Mn_0.4_O_3_ exceeds that (*T*_50_ = 134°C) of the noble metal Pd/Al_2_O_3_ catalyst.

## Conclusion

Conventionally, a special method has been required for the synthesis of *h*-REFeO_3_ due to its metastable nature; however, *h*-REFeO_3_ can now be easily synthesized by precisely controlling the calcination process even when employing the coprecipitation or sol-gel methods. *h*-REFeO_3_-based materials have been demonstrated to be applicable for catalyst materials, as well as magnetic or electronic materials. I believe that excellent catalyst materials will evolve from catalyst designs focusing on the unique crystal structure of *h*-REFeO_3_ or hexagonal-related materials (RE_n_Fe_n+1_O_(3n+1)_).

## Author Contributions

The author confirms being the sole contributor of this work and has approved it for publication.

### Conflict of Interest Statement

The author declares that the research was conducted in the absence of any commercial or financial relationships that could be construed as a potential conflict of interest.
